# Incidence of Common Preleukemic Gene Fusions in Umbilical Cord Blood in Slovak Population

**DOI:** 10.1371/journal.pone.0091116

**Published:** 2014-03-12

**Authors:** Milan Škorvaga, Ekaterina Nikitina, Miroslav Kubeš, Pavol Košík, Beata Gajdošechová, Michaela Leitnerová, Lucia Copáková, Igor Belyaev

**Affiliations:** 1 Department of Molecular Genetics, Cancer Research Institute, Slovak Academy of Sciences, Bratislava, Slovak Republic; 2 Laboratory of Oncovirology, Cancer Research Institute, Siberian Branch of the Russian Academy of Medical Sciences, Tomsk, Russian Federation; 3 Laboratory of R&D, Eurocord-Slovakia, Bratislava, Slovak Republic; 4 Department of Clinical Oncology, National Cancer Institute, Bratislava, Slovak Republic; Josep Carreras Leukaemia Research Institute, University of Barcelona, Spain

## Abstract

The first event in origination of many childhood leukemias is likely the presence of preleukemic clone (transformed hematopoietic stem/progenitor cells with preleukemic gene fusions (PGF)) in newborn. Thus, the screening of umbilical cord blood (UCB) for PGF may be of high importance for developing strategies for childhood leukemia prevention and treatment. However, the data on incidence of PGF in UCB are contradictive. We have compared multiplex polymerase chain reaction (PCR) and real-time quantitative PCR (RT qPCR) in neonates from Slovak National Birth Cohort. According to multiplex PCR, all 135 screened samples were negative for the most frequent PGF of B-lineage acute lymphoblastic leukemia (ALL) and acute myeloid leukemia (AML). To explore the prevalence of prognostically important TEL-AML1, MLL-AF4 and BCR-ABL (p190), 200 UCB were screened using RT qPCR. The initial screening showed an unexpectedly high incidence of studied PGF. The validation of selected samples in two laboratories confirmed approximately ¼ of UCB positive, resulting in ∼4% incidence of TEL-AML1, ∼6.25% incidence of BCR-ABL1 p190, and ∼0.75% frequency of MLL-AF4. In most cases, the PGF presented at very low level, about 1–5 copies per 10^5^ cells. We hypothesize that low PGF numbers reflect their relatively late origin and are likely to be eliminated in further development while higher number of PGF reflects earlier origination and may represent higher risk for leukemia.

## Introduction

Leukemia is a clonal disease arising from the transformation of a single cell, most probably a pluripotent hematopoietic stem cell (HSC) or a more mature progenitor cell [1]–[4]. The development of acute childhood leukemia is a multistep process driven by the accumulation of two kinds of genetic abnormalities. Primary abnormality or “first hit” represents an initiating event, often a chromosomal translocation generating a preleukemic gene fusion (PGF) with a novel activity impairing differentiation of HSC/progenitor cell. These gene fusions arise predominantly *in utero* during fetal hematopoiesis, producing a persistent but clinically covert preleukemic clone [2], [5], [6]. The preleukemic clone may convert to full leukemic transformation with acquisition of additional or secondary genetic changes, “second hit”, often point mutations, deletions, duplications.

At least 155 balanced rearrangements, most often chromosomal translocations, were found in acute lymphoblastic leukemia (ALL) [7]. The following four chromosomal translocations with corresponding PGF and frequencies are common in childhood ALL: t(12;21)(p13;q22) TEL-AML1 (24–26%), t(1;19)(q23;p13) E2A-PBX (5–6%), t(9;22)(q34;q11) BCR-ABL p190 (3–5%) and t(4;11)(q21;q23) MLL-AF4 (∼5%).The number of chromosomal translocations resulting in PGF is constantly growing with usage of new powerful screening approaches [8].

The acute leukemia is the most common childhood cancer in developed countries, accounting for one-third of all malignancies in this age group [9]. Childhood acute leukemia is a biologically diverse disease. ALL at 81% is the most frequent leukemia in Europe, followed by acute myeloid leukemia with 15%, and other three, markedly rare subgroups of chronic myeloid leukemia (CML) at 1.5%, unspecified (1.3%) and other specified leukemia (<0.5%) [10]. T-cell precursor ALL accounts for approximately 15% of childhood ALL, while B-cell precursor ALL accounts for major part [11]. About 1 child in every 2000 develops leukemia before the age of 15 years [12]. The peak incidence occurs very early in life, at around 1-3 years of age depending on the type of leukemia [10]. At later ages, the incidence drops off quite dramatically, with ∼90% lower incidence beyond the age of 15 [13]. The risk of a newborn being diagnosed with leukemia within the first 15 years of life is about 0.08% [10].

While childhood leukemia is treated with 85% long-term survival [14], depending on the phenotype and tumor genetics, most cured children face long-term side effects such as heart defects or chronic ailments, therefore, prevention and early detection of this disease is a goal [15]. In addition, the treatment outcome for relapsed ALL remains unsatisfactory (approximately 40% long-term survival) [16]. Human bone marrow (BM) has been considered as one of the main sources of HSC for both experimental and clinical applications. In past decades, human umbilical cord blood (UCB) has been regarded as an alternative source to BM cell transplantation and therapy because of its hematopoietic components. In particular, UCB cells are used for HSC transplantation to replace BM destroyed when treating leukemia. Human UCB is obtained after full-term delivery of the newborn from a sample that would inevitably be discarded.

Donor cell leukemia (DCL) is a rare but well-recognized complication that occurs after allogeneic HSC transplantation [17]. The DCL mortality remains very high. One possible mechanism for the development of DCL is that preleukemic clone was already present in the donor before transplant, but had remained undiagnosed. Thus, the screening of UCB for preleukemic clones may be of high importance for preventing DCL. The most efficient screening is based on analysis of PGF.

However, the data on incidence of PGF in UCB are contradictory. The widely accepted model of TEL-AML1^+^ leukemogenesis suggested that the initiating genetic event, i.e. the t(12;21) chromosomal translocation resulting into TEL-AML1 fusion occurs at relatively high proportion (∼1%) of newborns [5], [6], [18]–[20]. Taking into consideration the cumulative incidence of TEL-AML1^+^ ALL in children (1∶10,000, i.e. 0.01%), it predicts that only 1 of 100 newborns harboring detectable TEL-AML1 transcripts are destined to develop ALL [21]. This scenario significantly limits the utility of UCB screening for the presence of preleukemic clones. Recently, a Danish group has challenged this scenario, providing evidence that the proportion of newborns with detectable TEL-AML1 transcripts may actually be much lower (<0.01%) implying that a high proportion of infants, up to 100%, born with detectable TEL-AML1 fusion will eventually develop TEL-AML1^+^ ALL [22]. In this scenario, the UCB screening could be of importance in attempts to prevent the development of ALL in TEL-AML1^+^ children during preleukemic phase and prevent usage of such samples for allogeneic stem cell transplantation.

The conflicting results on incidence of preleukemic clones in UCB might be caused by (i) differences in methods of screening and confirmation, or (ii) different incidences between geographical regions screened in relevant studies.

In this study, we have compared two approaches: (i) multiplex polymerase chain reaction (PCR) [23], and (ii) real-time quantitative PCR (RT qPCR) [24] in screening the PGF in UCB from Slovak National Birth Cohort. According to multiplex PCR, all 135 screened samples were negative for the most frequent PGF of B-lineage ALL: TEL-AML1, E2A-PBX, MLL-AF4, and BCR-ABL (p190) and for the most frequent PGF of acute myeloid leukemia (AML): AML-ETO, PML-RARA, and CBFβ-MYH11. To explore the prevalence of most important prognostic fusion genes TEL-AML1, MLL-AF4 and BCR-ABL (p190), 200 UCB were screened for PGF transcripts using more sensitive RT qPCR.

## Materials and Methods

### Ethics statement

This study has been approved by the Ethics Committee of Children's Hospital in Bratislava. Human UCB samples were obtained with written parental informed consent. The Ethics Committee has approved this consent procedure.

### Isolation of RNA from UCB samples

UCB was syringed out of the placenta through the umbilical cord after the cord has been detached from the newborn. All 200 newborns were born healthy after full-term pregnancies. Mononuclear cells (MNC) were isolated from 80–100 ml of UCB, within 24 hours after birth by the standard gradient centrifugation using LymphoSep™ (MP Biomedicals, USA). Number of cells was assessed using autohematology analyzer (Mindray, BC-3000plus, China). Isolated UCB MNC pellets were then shocked frozen in liquid nitrogen. Each cell pellet, containing ∼10^7^ MNC and provided in at least triplicates, was cryopreserved by a controlled rate freezer and stored in liquid nitrogen.

For RNA isolation, a single cell pellet was thawed and total RNA was isolated with RNAzol (Research Molecular Center, Ohio, USA) using standard protocol recommended by manufacturer. The concentration and purity of isolated RNA was measured by Nanodrop N-1000 instrument (Thermo Scientific, Delaware, USA).

To assess the suitability of RNA isolation method, the integrity of eight RNA samples, isolated by RNAzol method, was measured on Agilent 2100 Bioanalyzer (Agilent Technologies, Inc., California, USA) and their RIN (RNA integrity number) was estimated. RIN data are shown in Table 1. All RIN exceeded threshold for reliable RT qPCR results: RIN > 4.1. The average RIN value of selected RNA samples was very high, reaching ∼8.7, and suggesting that RNAzol method for isolation of total RNA from UCB MNC is highly appropriate. Subsequently, the integrity of RNAs was determined by running samples on 1.5% denaturing agarose gel and visual assessment of intensity of 28S and 18S rRNA bands. The suitability of RNA for subsequent PCR screening was estimated either by PCR amplification of cDNA using 18S rRNA specific primers (for multiplex PCR) or by quantification of control *ABL* gene (c-*ABL*) following the standardized RT qPCR protocol [24]. RNA was stored at −80°C.

**Table 1:** RNA integrity number (RIN) of selected RNA samples isolated from UCB MNC by the RNAzol method.

Proband No.	RIN
43	9.5
44	9.1
47	9.3
48	9.1
49	8.5
53	8.8
55	9.1
56	6.5

The common fusion transcripts associated with acute childhood leukemia were analyzed by two PCR techniques: (a) multiplex reverse-transcription (RT) PCR [23], (b) real-time quantitative PCR (RT qPCR) [24]. In addition, some of the positive samples were verified by a nested PCR. All the precautionary measures we have been taking against contamination are given in Text S1.

### Multiplex RT-PCR and nested PCR

cDNA used as a template in the PCR, was reverse transcribed from 1 µg of total RNA using 1 mM dNTP mix, random hexamer and oligo(dT)_18_ primer 5 µM each, 20U RNAse inhibitor and 200U RevertAid H^−^ Reverse Transcriptase following manufacturer's protocol (Thermo Scientific, St. Leon-Rot, Germany).

We used multiplex RT-PCR designed by *Pakakasama et al.* [23]. In brief, the common fusion transcripts of chromosomal translocations were divided into two panels. Panel A allows identification of the most frequent fusion transcripts of B-lineage ALL, including TEL-AML1, E2A-PBX, MLL-AF4, and BCR-ABL (p190), while panel B is set for AML consisting of AML-ETO, PML-RARA, and CBFβ-MYH11. The primers for each panel as well as for simplex and nested PCRs were synthesized by Integrated DNA Technologies (IDT, Coralville, Iowa, USA) and designed according to *van Dongen et al.* [25]. We assumed that a sample identified as positive by multiplex PCR appearing as a specific DNA band on gel electrophoresis, is to be confirmed by simplex PCR using [A+B] primers for an individual fusion transcript and subsequently, by nested PCR with internal [C+D] primers. A 15-µl aliquot of multiplex PCR (total reaction volume of 25 µl) was visualized on 1% agarose gel stained with EtBr in 0.5× TBE running buffer. The screening with multiplex PCR was performed in single reactions.

### Real-time quantitative PCR

The RT qPCR contained 2 µl cDNA (100 ng RNA equivalent), 300 nM each primer, 200 nM probe (5′-fluorophore was FAM, 3′-quencher was BHQ1; synthesized by Merck), and TaqMan universal PCR master mix from Applied Biosystems. The primers and probes were synthesized by VBC-Biotech (Wien, Austria) and designed according to *Gabert et al.* [24]. The plasmid standards with individual fusion genes subcloned into PCR II TOPO vector were from Ipsogen (Qiagen, Marseille, France). We also prepared own plasmid standards, by religating ∼10 pg of commercial pDNA, amplifying in *E.coli* and linearizing with a restriction endonuclease. At Cancer Research Institute (CRI), Bratislava, the RT qPCR were performed either on RotorGene 2000 or BioRad CFX96 instrument following the protocol by *Gabert et al.* [24]. Each sample was analyzed at least in triplicates. Samples were regarded as positive for a particular rearrangement if a fusion transcript was present in at least one reaction. For verification of CRI results, twenty selected samples in the form of 10^7^ MNC frozen pellets were processed in a certified laboratory at National Cancer Institute (NCI), Bratislava, in an identical manner as described above, with three minor exceptions, namely (i) total RNA was isolated with Trizol (Invitrogen), (ii) the 3′-quencher was TAMRA and (iii) RT qPCR instrument was RotorGene 3000. In CRI laboratory, some of the positive samples were further verified by nested PCR. Another set of 15 selected samples was verified in CRI with BioRad CFX96 instrument.

### PCR sensitivity

To assess and compare the sensitivity level of PCR in our hands, we performed sensitivity tests for multiplex RT-PCR, RT qPCR, and nested PCR. For each type of PCR, the test reactions were run at the same conditions as analyzed samples, with following exceptions: (i) as a template each reaction contained defined amount of a commercial standard plasmid (from 1 to 100 copies *per* reaction) and (ii) cDNA mix (1 or 2 µl of final cDNA which has been tested negative in previous PCR analyses). Each sample was replicated 5 times.

## Results

The present study examined the incidence of common fusion transcripts associated with ALL in children in UCB from healthy neonates in Slovak population. The available data on incidence of preleukemic clones in UCB from healthy individuals which are highly related to our study is very contradictory and there has recently been a wide discussion about the appropriate methodological approaches to resolve this puzzle [20]–[22], [26]. Our work compares two major PCR techniques, commonly used in this type of analysis, namely multiplex RT-PCR and RT qPCR.

For this type of screening, it is necessary to assess the sensitivity of the detection methods as the sensitivity of PCR methods is expected to vary across different laboratories. To determine the sensitivity of used PCR techniques, a defined number of copies of standard plasmid, containing a specific fusion gene, were mixed with ‘negative’ cDNA, i.e. cDNA tested negative in all PCR methods applied. The results of PCR sensitivity are shown in Tables 2, 3.

**Table 2:** Multiplex PCR sensitivity assessed as ratio of positive reactions to all reactions at standard plasmid level.

Copies/PCR	TEL-AML1	MLL-AF4	BCR-ABL p190
10	0.6	0.8	0.4
50	1	1	0.8
100	1	1	1

**Table 3:** RT qPCR and Nested PCR sensitivity assessed as ratio of positive reactions to all reactions at standard plasmid level.

	TEL-AML1		MLL-AF4		BCR-ABL p190	
Copies/PCR	RT qPCR	Nested PCR	RT qPCR	Nested PCR	RT qPCR	Nested PCR
1	0.6	0	0.2	0.2	0.4	0.6
3	0.8	0.6	-	0.4	0.6	0.6
5	0.6	0.8	1	0.8	1	0.8
10	1	1	1	1	1	1

Our further calculations are based on experimental assessment of yield of RNA per UCB MNC and presumption that a single MNC contains ∼1 pg RNA. Total RNA was isolated from 10^7^ MNC, yielding ∼10 µg RNA. 1 µg total RNA was used for cDNA synthesis, corresponding to ∼10^6^ cells. 1/10 of final cDNA for RT qPCR (1/20 for multiplex or nested PCR) was utilized in PCR reactions, which is equivalent to ∼10^5^ (∼5×10^4^) cells. On the basis of these assumptions we extrapolate the sensitivity level for each tested PCR approach. The sensitivity rate of our multiplex PCR accomplished ∼20–100 copies/10^5^ cells [∼0.2–1×10^−3^] (Table 2). In comparison, the sensitivity of both RT qPCR and nested PCR is much higher, reaching the level of about 1–3 copies/10^5^ cells [∼1–3×10^−5^] (Table 3).

Based on previously published data of Mori *et al.* suggesting that about 1% of newborns were positive for TEL-AML1 and the frequency of positive cells was calculated to be between 10^−3^ and 10^−4^ [6], we assumed that sensitivity of multiplex PCR may be appropriate to reveal preleukemic clones in UCB. Using multiplex PCR, we analyzed samples from 135 probands. All probands were negative for all three examined fusion transcripts at the defined sensitivity of ∼0.2–1×10^−3^ and about 50% sensitivity at 10^−4^. In contrast to the data by Mori *et al*. [6], who used nested PCR for screening of TEL-AML1, multiplex PCR did not detect any PGF in UCB of 135 probands (data are not shown). Lower sensitivity level of multiplex PCR relative to expected number of copies positive for a fusion transcript in UCB samples might be reason for negative results obtained with multiplex PCR. Thus, we used RT qPCR which has higher sensitivity. Out of 135 probands tested negative by multiplex PCR, 15, 15 and 1 probands were found positive by RT qPCR for TEL-AML1, BCR-ABL p190 and MLL-AF4, respectively. With RT qPCR, 16% (32/200) of cord blood samples were tested positive for TEL-AML1, 3% (6/200) positive for MLL-AF4, and 25% (50/200) positive for BCR-ABL p190, at the sensitivity level of approx. 1–3×10^−5^.

**Figure 1:**
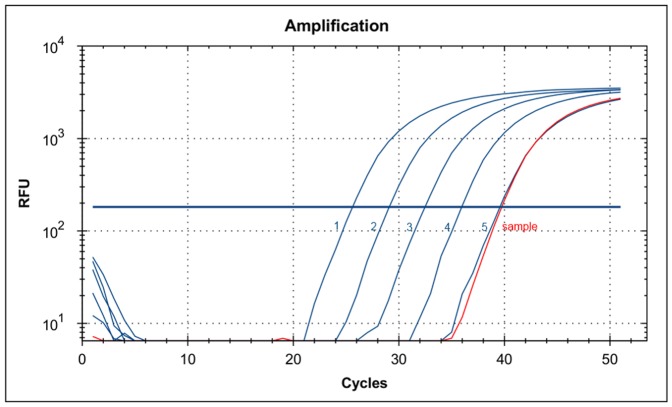
RT qPCR profiles of selected positive probands acquired by CRI. RT qPCR profiles of BCR-ABL p190 plasmid standards: 20000, 2000, 200, 20, 2 copies *per* reaction, blue lines from 1 to 5, respectively, and a sample from proband #297, red line. The efficiency of RT qPCR reached 96.3% and R^2^ value was 0.998. Proband #297 tested positive was calculated to contain 1 copy (C_t_ = 39.68) of BCR-ABL p190 fusion transcript *per* ∼100,000 cells.

It is interesting that in majority of positive samples only one out of three reactions gave positive signal suggesting very low number of preleukemic cells in UCB of newborns (Figure S1). Indeed in most positive samples the number of preleukemic cells were assessed to be within 1–5 copies *per* 10^5^ cells, while in a few probands we have found higher numbers, for example probands #140 and #144 were both tested 3/3 positive with 17, 9, 15 and 44, 50, 3 copies of BCR-ABL fusion transcripts, respectively. We found our data surprising by two reasons. First, determined by us incidence rate was too high as compared to ≤1% incidence expected from recently published models [21]. Second, TEL-AML1 was anticipated to be more frequent type of rearrangement than BCR-ABL. Therefore, 20 positive samples (as MNC pellets) were verified in a certified laboratory at NCI. This laboratory employs the same RT qPCR technique as CRI laboratory based on publication of Gabert *et al* [24]. Out of 20 samples, only five fusion transcripts were confirmed by RT qPCR analysis at NCI (Table 4). Figure 1 represents an example of RT qPCR profiles of positive probands. Critical parameters of RT qPCR analysis of samples being validated, including C_t_ and copy number for c-Abl control gene and particular fusion gene as well as the reaction efficiencies and R^2^ values are given in Tables S1, S2, S3.

**Table 4:** Comparison of RT qPCR results for selected samples analysed at Cancer Research Institute (CRI) and National Cancer Institute (NCI) (replicated positive samples are shown in bold).

		TEL-AML1		MLL-AF4		BCR-ABL p190	
No.	Proband	CRI	NCI	CRI	NCI	CRI	NCI
1.	29	1/3	0/3	0/3	0/3	1/3	0/3
2.	**41**	1/3	0/3	0/3	0/3	**1/3**	**1/3**
3.	**52**	0/3	0/3	0/3	1/3	**1/3**	**1/3**
4.	68	0/3	1/3	1/3	0/3	0/3	0/3
5.	84	0/3	0/3	0/3	0/3	1/3	0/3
6.	139	1/3	0/3	0/3	1/3	1/3	0/3
7.	140	2/3	0/3	0/3	0/3	3/3	0/3
8.	**141**	0/3	0/3	0/3	0/3	**2/3**	**1/3**
9	144	3/3	0/1	0/3	0/1	2/3	0/3
10.	**145**	0/3	1/3	1/3	0/3	**2/3**	**1/3**
11.	146	0/3	0/3	0/3	0/3	2/3	0/3
12.	150	0/3	0/3	0/3	0/1	1/3	0/1
13.	163	0/3	0/3	0/3	0/3	1/3	0/3
14.	191		0/3	0/3	0/3	1/3	0/3
15.	203	0/3	0/1	0/3	0/3	2/3	0/3
16.	**206**	0/3	0/3	0/3	0/3	**1/3**	**1/3**
17.	214	0/3	0/3	0/3	0/3	0/3	0/3
18.	215	1/3	0/3	0/3	0/3	3/3	0/3
19.	216	1/3	0/3	0/3	0/3	2/3	0/3
20.	217	0/3	0/3	0/3	0/3	2/3	0/3

All seven selected UCB samples tested positive for TEL-AML1 in CRI laboratory were found negative by a certified NCI laboratory. The negativity of 13 selected samples for this translocation was confirmed by NCI. Out of 18 BCR-ABL p190 positive samples, only five were confirmed at NCI. Two UCB samples were tested negative for this translocation in both laboratories. Single MLL-AF4 positive sample was not validated, in contrary out of 18 negative samples two were detected as positive at NCI. In total, out of 32 samples tested negative in CRI laboratory, 29 were validated by NCI, resulting into ∼90% validation rate of negative samples. Overall, these data show a certain discrepancy in the fusion transcript detection between the two laboratories, which, however, does not exceed discrepancies between other laboratories [6], [22]. The major difference in processing samples between CRI laboratory and reference NCI laboratory was in the method of isolation of total RNA from MNC, obtained from UCB, using RNAzol and TRIzol methods, respectively. It has been shown that RNA isolated by both these methods exhibited comparable results in quantitative competitive RT-PCR amplification of the *ABL* gene [27]. In addition, RNA isolated by RNAzol was DNA-free, in a slightly higher yield than RNA isolated by TRIzol where major contaminants with genomic DNA were observed. The TRIzol method is routinely used for isolation of RNA from patient samples at NCI. The presence of genomic DNA in RNA sample should not have a fundamental impact, if any, in our assay because the templates for cDNA synthesis are RNA fusion transcripts, not genomic DNA. However, a significant contamination of total RNA with genomic DNA might have a profound effect on a correct estimation of RNA concentration in the sample resulting into lower than optimal amount of RNA template for cDNA synthesis. Second difference was 3′-quencher in TaqMan probe, being BHQ-1 and TAMRA in CRI and NCI, respectively. The major difference between BHQ-1 and TAMRA is that the former is a dark quencher which re-emits its energy as heat rather than light, while the latter fluoresces. It has been shown that non-specific background fluorescence of TAMRA-quenched probe might reduce sensitivity of a TaqMan assay [28]. Data also suggest that use of BHQ-1 resulted in 1.2-2.8-fold decrease of intra-assay variability as compared to use of TAMRA [28]. In addition, the use of different quenchers can have also an impact on stability of duplex template-probe, e.g. BHQ-1 was shown to have higher stability effect on probe-target DNA duplex than TAMRA. Majority of RT qPCR assays in CRI were performed on RotorGene 2000 while NCI uses RotorGene 3000 – an upgraded instrument with better software and wider utility of different fluorescent dyes/quenchers. However, both these instruments are very similar, reliable and accurate. If we take into account all the above mentioned aspects we may assume that the sensitivity of RT qPCR might be slightly higher at CRI than that at NCI. A similar assumption might by applied also for BioRad CFX96 since with this instrument we achieved similar validation rate as that at NCI. It is important to stress that in our case when the amount of pre-leukemic cells containing a fusion transcript in most of UCB is on threshold of the sensitivity of RT qPCR method, only a marginal difference in the sensitivity might have significant effects on the results.

The high frequencies of fusion transcripts in our sample collection lead us to verify whether these positives are false-positive e.g. due to cross-contamination of the components of PCR reaction, i.e. primers, probes, buffers, water, master mix. Therefore, we performed a RT qPCR experiment in a 96-well format (on BioRad CFX96 instrument) with only non template controls (NTC), water and plasmid standards, and the result was that all NTC's as well as water samples were clearly negative. This finding along with fact that NTC ran in triplicates were found negative in all RT QPCR experiments suggest that the positivity of UCB samples as detected in our laboratory was probably not caused by contamination during PCR. However, other sources of contamination, e.g. during isolation of MNC from UCB, RNA isolation, and cDNA synthesis cannot be excluded, although we followed strict precautionary measures in attempt to avoid contamination (Text S1).

In order to further validate our data, we re-analyzed another set of 15 samples, originally tested positive for BCR-ABL p190 translocation when analyzed on RotorGene 2000. We started with isolation of total RNA by RNAzol method from never opened tubes with MNC pellets and all the steps were identical with those used in the first screening, except that the analysis was performed on BioRad CFX96 instrument. These data are shown in Table 5 and critical parameters of RT qPCR analysis are given in Table S4. Our data show that the BCR-ABL p190 positivity was confirmed in 4 out of 15, i.e. ∼26.6% samples. In summary, we achieved similar verification rate at CRI laboratory as reference NCI laboratory, approx. 1/4. If this validation rate is applied to frequency of all three fusion genes screened in our set, rough estimated incidence of studied fusion genes in UCB in Slovak population would be as follows (Table 6).

**Table 5:** Comparison of 1^st^ and 2^nd^ screening of selected 15 samples analyzed by RT qPCR (replicated positive samples are shown in bold).

		1^st^ screening	2^nd^ screening
No.	Proband	(RotorGene 2000)	(BioRad CFX96)
1.	44	1/3	0/3
2.	166	1/3	0/3
3.	167	1/3	0/3
4.	173	1/3	0/3
5.	**196**	**1/3**	**1/3**
6.	200	1/3	0/3
7.	250	1/3	0/3
8.	251	1/3	0/3
9	257	1/3	0/3
10.	259	2/3	0/3
11.	262	1/3	0/3
12.	265	1/3	0/3
13.	**297**	**2/3**	**1/3**
14.	**307**	**1/3**	**2/3**
15.	**308**	**1/3**	**2/3**

**Table 6:** Estimated incidences of three prognostically important fusion genes in Slovak population.

	1st screening	After verification
Preleukemic gene fusions	(CRI, RotorGene 2000)	(NCI/CRI, RotorGene 3000/BioRad CFX96)
TEL-AML1	16%	4%
BCR-ABL p190	25%	6.25%
MLL-AF4	3%	0.75%

## Discussion

One way to determine susceptibility to childhood leukemia is to search for preleukemic clones by analysis of leukemia-specific chromosomal translocations in hematopoietic stem/progenitor cells of umbilical cord blood. The major objective of this work was to estimate prevalence of prognostically important leukemic gene fusions in Slovak population.

Using RT qPCR with calculated sensitivity of 1–3×10^−5^ and after applying validation rate of 1/4 we estimated frequencies of UCBs positive for TEL-AML1 at 4%, BCR-ABL p190 at 6% and MLL-AF4 at 0.75%. These data were highly surprising with respect to on-going discussion on the subject whether the frequency of TEL-AML1 preleukemic clones in UCB is 1% (model A) or 0.01% (model B) [21].

The data supporting model A come from studies more than 10-years old showing ∼1.5% (1/67) UCBs tested positive for TEL-AML1 by nested PCR [29] and mainly by Mori's data demonstrating ∼1% (6/567) UCBs positive for this fusion at cell levels of 10^−3^ to 10^−4^ [6]. However, the data of Mori *et al*. were not confirmed by more recent work of Danish group suggesting much lower incidence of TEL-AML1 fusion transcripts at birth, namely ∼0.01% [22]. So far, these data have not been supported by other studies.

In general, the data on incidence of all common fusion transcripts in UCB as well as other cell types are highly inconsistent. In contrast to above mentioned data, several reports have been published showing much higher frequency of fusion transcripts in neonates as well as in healthy adults. For example, Uckun *et al* [30] reported relatively very high incidence of MLL-AF4 fusion transcripts in fetal liver (38%), fetal BM (25%) and neonatal BM (17%) using nested PCR with ∼10^−4^ sensitivity. However, these frequencies were not confirmed by other groups which failed to identify MLL-AF4 transcripts by RT-PCR, e.g. 0/130 in UCB [31], or 0/60 in UCB and 0/8 in fetal liver [32], while Mori et al. showed ∼0.2% (1/496) UCB positive [6].

BCR-ABL p190 is found in about 3–5% of ALL cases, while TEL-AML1 is a most frequent PGF, about 24–26% ALL. Thus, the fact that BCR-ABL p190 (and not TEL-AML1) is the most frequent fusion transcript is unexpected result of our study. However, there are several studies showing relatively high incidence of this fusion gene in healthy individuals. Using a 2-step RT-PCR with total sensitivity of up to 10^−8^, Bose *et al* [33] identified the BCR-ABL p190 (e1a2 junction) in circulating leukocytes in 11 out of 16 (∼69%) healthy adults. In addition, they showed that 7 out of 7 screened human hematopoietic cell lines were tested positive for BCR-ABL p190. Interestingly, several fusion transcripts tested as positive in leukocytes contained ‘wrong’ junction between BCR and ABL exons, resulting into a non-functional fusion protein representing false positivity. As it was demonstrated later, a premature termination of transcripts participating in intergenic trans-splicing events in the absence of corresponding chromosomal translocation may represent another source of positivity [34]. More recently, Song *et al* [19] demonstrated 42% (21/50) incidence of BCR-ABL p190 in UCBs using both nested and RT qPCR with identical sensitivity of 10^−4^, and this fusion transcript was detected in 74% (59/80) of healthy individuals.

The quantitation analysis of our data showed that the initial copy number of a fusion transcript was low in the majority of analyzed samples corresponding to < 10 copies *per* 100,000 cells. We also observed that in most cases only 1 reaction out of triplicate was recorded positive (Figure S1). These data may indicate that the amount of preleukemic cells with a common fusion transcript in vast majority of UCB is on the threshold of the sensitivity of the RT qPCR/nested PCR methods used and this may be the major factor responsible for low validation rate.

Our data in combination with conflicting findings from other laboratories lead us to following idea. If the frequencies of PGF detected in UCB samples of healthy newborns were really so high, i.e. ∼4% TEL-AML1, ∼6% BCR-ABL p190 and 0.75% MLL-AF4, with a defined frequency of children leukemia (1∶10,000), it would suggest that the presence of PGF in a newborn is not as important as the number of HSC/progenitor cells bearing a fusion transcript in the particular UCB which depends on the time during fetal development when this chromosomal rearrangement arose. Therefore, we suggest that the crucial parameter would be the level of signal, i.e. the amount of cells containing the chromosomal translocation (PGF), reflecting the time of its generation. In case, when only a single or few HSC/progenitor cells are positive, this might not represent an increased risk for leukemia development in the carrier of the PGF. In contrast, when this fusion gene is generated and expressed very early during fetal hematopoiesis, inflicting in this way more cells, then this might cause increased risk for its carrier and DCL following cord blood transplantation [35]. We suggest that a precise quantification of positive signal in appropriate (leukemogenic) cell population should be used instead of “signal positivity” as a measure of the risk of the preleukemic clone present in UCB for leukemia.

It is interesting to note a very low signal in all six samples in our study tested positive for MLL-AF4 (1/3 positivity, ≤2 copies/sample). If our assumption mentioned above is correct, then these data suggest that t(4;11) translocation in these probands may be generated later during fetal development, most probably in irrelevant cells, thus not representing a higher risk for their carriers to develop an aggressive MLL-AF4^+^ B-ALL at or shortly after birth. There is no doubt that at least MLL-AF4 and TEL-AML1 originate prenatally *in utero* during embryonic/fetal development [2], [36], [37]. One of the crucial questions is what type of cells (in hierarchy and ontogenesis) is the target for the 1^st^ genetic hit, which results in chromosomal translocation. Recent data suggest that the target cells for initial oncogenic hit in fetal MLL-AF4^+^ may be mesodermal pre-hematopoietic cells or hemangioblasts [38], [39] rather than more committed HSC/progenitor cells generally assumed as target cells for pediatric TEL-AML1^+^ B-ALL. It means that in infant B-ALL the initial genetic hit must take place in limited targets in a limited time, probably in first few months of embryogenesis as opposed to pediatric B-ALL where much higher numbers of target cells and longer period for initial hit are ‘provided’. Also the vulnerability/susceptibility of respective target cells may be different. Despite the fact that the overall frequency of infant MLL-AF4^+^ is several fold lower when compared to pediatric TEL-AML1^+^, we may expect a higher MLL-AF4 than TEL-AML1 signal in corresponding UCB samples tested positive because MLL-AF4 arise earlier and inflict more cells. A precise quantification of positive signal and immunophenotyping of PGF-positive cells in backtracked UCB from neonates with infant MLL-AF4^+^ *versus* TEL-AML1^+^ B-ALL would give an answer whether our assumptions are correct and whether UCB screening for the presence of MLL-AF4 is relevant. Recent data suggested a possibility that UCB HSC/progenitor cells may not represent appropriate target for MLL-AF4 to induce transformation [40]. Bueno and colleagues created an elegant hESC model allowing to study the regulation of human embryonic hematopoiesis [37], [41], [42]. Our data support the use of this system which can help to define the timing of the initial oncogenic lesion and the developmental position of the target cell within the differentiation cascade as they have a profound influence on the final leukemogenic phenotype. In addition, the identification of secondary prenatal and/or postnatal leukemogenic events could help us to better understand the molecular mechanisms of the processes which are essential for the transformation of a preleukemic clone into an overt disease. This in turn may help to discover new therapy strategies and improve prognosis of leukemic patients.

## Supporting Information

Figure S1 Positivity fraction *per* triplicate.
(TIF)

Table S1 Comparison of RT-qPCR results for selected samples analyzed at Cancer Research Institute (CRI) and National Cancer Institute (NCI) – TEL-AML1 analysis.
(DOCX)

Table S2 Comparison of RT-qPCR results for selected samples analyzed at Cancer Research Institute (CRI) and National Cancer Institute (NCI) – MLL-AF4 analysis.
(DOCX)

Table S3 Comparison of RT-qPCR results for selected samples analyzed at Cancer Research Institute (CRI) and National Cancer Institute (NCI) – BCR-ABL p190 analysis (replicated positive samples are shown in bold).
(DOCX)

Table S4 Comparison of 1^st^ and 2^nd^ screening of selected 15 samples analyzed by RT-qPCR at CRI (replicated positive samples are shown in bold).
(DOCX)

Text S1 Precautionary measures against contamination.
(DOCX)
