# Supplementary material for: Incidence of Common Preleukemic Gene Fusions in Umbilical Cord Blood in Slovak Population
Source: PLoS One. 2014 Mar 12;9(3):e91116. doi: 10.1371/journal.pone.0091116 (PMC3951330; doi:10.1371/journal.pone.0091116)
Supplement: Table S1 — Comparison of RT-qPCR results for selected samples analyzed at Cancer Research Institute (CRI) and National Cancer Institute (NCI) – TEL-AML1 analysis. (DOCX) [file pone.0091116.s002.docx]

### Table S1. Comparison of RT-qPCR results for selected samples analyzed at Cancer Research Institute (CRI) and National Cancer Institute (NCI) – TEL-AML1 analysis.

|  |  | **CRI** | | | | **NCI** | | | |
| --- | --- | --- | --- | --- | --- | --- | --- | --- | --- |
| **No.** | **Proband** | **Ct [c-ABL]** | **Positivity** | **Ct [TEL-AML1]** | **Copies** | **Ct [c-ABL]** | **Positivity** | **Ct [TEL-AML1]** | **Copies** |
| 1. | 29 | 25.46 | 1/3 | 39.93 | 1 | 23.98 | 0/3 | - | - |
| 2. | 41 | 26.44 | 1/3 | 35.71 | 3 | 23.51 | 0/3 | - | - |
| 3. | 52 | 24.63 | 0/3 | - | - | 23.14 | 0/3 | - | - |
| 4. | 68 | 25.8 | 0/3 | - | - | 22.56 | 0/3 | 44.71 | 0 |
| 5. | 84 | 23.98 | 0/3 | - | - | 23.68 | 0/3 | - | - |
| 6. | 139 | 23.78 | 1/3 | 34.34 | 15 | 23.15 | 0/3 | - | - |
| 7. | 140 | 24.21 | 3/3 | 33.37 | 22 | 22.73 | 0/3 | - | - |
|  |  |  |  | 32.56 | 32 |  |  |  |  |
|  |  |  |  | 33.76 | 19 |  |  |  |  |
| 8. | 141 | 23.97 | 0/3 | - | - | 22.30 | 0/3 | - | - |
| 9 | 144 | 24.74 | 3/3 | 34.83 | 4 | 24.53 | 0/1 | - | - |
|  |  |  |  | 36.28 | 2 |  |  |  |  |
|  |  |  |  | 36.46 | 1 |  |  |  |  |
| 10. | 145 | 24.5 | 0/3 | - | - | 22.47 | 0/3 | 44.88 | 0 |
| 11. | 146 | 24.45 | 0/3 | - | - | 23.22 | 0/3 | - | - |
| 12. | 150 | 26.6 | 0/3 | - | - | 25.19 | 0/3 | - | - |
| 13. | 163 | - | 0/3 | - | - | 22.78 | 0/3 | - | - |
| 14. | 191 | 23.75 | 0/3 | - | - | 22.98 | 0/3 | - | - |
| 15. | 203 | 26.7 | 0/3 | - | - | 23.41 | 0/1 | - | - |
| 16. | 206 | 26.1 | 0/3 | - | - | 23.12 | 0/3 | - | - |
| 17. | 214 | 26.48 | 0/3 | - | - | 23.83 | 0/3 | - | - |
| 18. | 215 | 26.42 | 1/3 | 37.64 | 7 | 23.31 | 0/3 | - | - |
| 19. | 216 | 25.85 | 1/3 | 37.93 | 6 | 23.77 | 0/3 | - | - |
| 20. | 217 | 25.58 | 0/3 | - | - | 23.1 | 0/3 | - | - |
| Efficiency | | 92.0% | | 92.7% | | 113.9% | | 100.9% | |
| R2 value | | 0.99035 | | 0.99820 | | 1.0000 | | 0.99728 | |
