# Supplementary material for: Incidence of Common Preleukemic Gene Fusions in Umbilical Cord Blood in Slovak Population
Source: PLoS One. 2014 Mar 12;9(3):e91116. doi: 10.1371/journal.pone.0091116 (PMC3951330; doi:10.1371/journal.pone.0091116)
Supplement: Table S3 — Comparison of RT-qPCR results for selected samples analyzed at Cancer Research Institute (CRI) and National Cancer Institute (NCI) – BCR-ABL p190 analysis (replicated positive samples are shown in bold). (DOCX) [file pone.0091116.s004.docx]

### Table S3. Comparison of RT-qPCR results for selected samples analyzed at Cancer Research Institute (CRI) and National Cancer Institute (NCI) – BCR-ABL p190 analysis (replicated positive samples are shown in bold).

|  |  | **CRI** | | | | **NCI** | | | |
| --- | --- | --- | --- | --- | --- | --- | --- | --- | --- |
| **No.** | **Proband** | **Ct [c-ABL]** | **Positivity** | **Ct [p190]** | **Copies** | **Ct [c-ABL]** | **Positivity** | **Ct [p190]** | **Copies** |
| 1. | 29 | 25.46 | 1/3 | 36.54 | 4 | 23.98 | 0/3 | - | - |
| 2. | **41** | 26.44 | **1/3** | 36.27 | 1 | 23.51 | **1/3** | 38.28 | 1 |
| 3. | **52** | 24.63 | **1/3** | 37.02 | 2 | 23.14 | **1/3** | 39.26 | 1 |
| 4. | 68 | 25.8 | 0/3 | - | - | 22.56 | 0/3 | - | - |
| 5. | 84 | 23.98 | 1/3 | 37.32 | 1 | 23.68 | 0/3 | - | - |
| 6. | 139 | 23.78 | 1/3 | 36.27 | 8 | 23.15 | 0/3 | - | - |
| 7. | 140 | 24.21 | 3/3 | 34.43 | 17 | 22.73 | 0/3 | - | - |
|  |  |  |  | 35.72 | 9 |  |  |  |  |
|  |  |  |  | 34.72 | 15 |  |  |  |  |
| 8. | **141** | 23.97 | **2/3** | 36.82 | 6 | 22.3 | **1/3** | 38.01 | 1 |
|  |  |  |  | 36.63 | 7 |  |  |  |  |
| 9 | 144 | 24.74 | 3/3 | 32.43 | 44 | 24.53 | 0/1 | - | - |
|  |  |  |  | 32.14 | 50 |  |  |  |  |
|  |  |  |  | 35.70 | 3 |  |  |  |  |
| 10. | **145** | 24.5 | **2/3** | 33.30 | 29 | 22.47 | **1/3** | 38.54 | 1 |
|  |  |  |  | 32.28 | 46 |  |  |  |  |
| 11. | 146 | 24.45 | 2/3 | 33.32 | 17 | 23.22 | 0/3 | - | - |
|  |  |  |  | 34.45 | 9 |  |  |  |  |
| 12. | 150 | 26.6 | 1/3 | 37.87 | 1 | 25.19 | 0/1 | - | - |
| 13. | 163 | - | 1/3 | 39.54 | 2 | 22.78 | 0/3 | - | - |
| 14. | 191 | 23.75 | 1/3 | 37.43 | 6 | 22.98 | 0/3 | - | - |
| 15. | 203 | 26.7 | 2/3 | 36.42 | 2 | 23.41 | 0/3 | - | - |
|  |  |  |  | 36.64 | 2 |  |  |  |  |
| 16. | **206** | 26.1 | **1/3** | 33.59 | 12 | 23.12 | **1/3** | 39.41 | 1 |
| 17. | 214 | 26.48 | 0/3 | - | - | 23.83 | 0/3 | - | - |
| 18. | 215 | 26.42 | 3/3 | 36.06 | 2 | 23.31 | 0/3 | - | - |
|  |  |  |  | 37.11 | 2 |  |  |  |  |
|  |  |  |  | 37.09 | 2 |  |  |  |  |
| 19. | 216 | 25.85 | 2/3 | 35.95 | 4 | 23.77 | 0/3 | - | - |
|  |  |  |  | 35.73 | 4 |  |  |  |  |
| 20. | 217 | 25.58 | 2/3 | 35.52 | 3 | 23.1 | 0/3 | - | - |
|  |  |  |  | 35.93 | 3 |  |  |  |  |
| Efficiency | | 92.0% | | 90.0% | | 113.9% | | 98.3% | |
| R2 value | | 0.0997 | | 0.9939 | | 1.000 | | 0.99952 | |
