# Supplementary material for: Incidence of Common Preleukemic Gene Fusions in Umbilical Cord Blood in Slovak Population
Source: PLoS One. 2014 Mar 12;9(3):e91116. doi: 10.1371/journal.pone.0091116 (PMC3951330; doi:10.1371/journal.pone.0091116)
Supplement: Table S4 — Comparison of 1st and 2nd screening of selected 15 samples analyzed by RT-qPCR at CRI (replicated positive samples are shown in bold). (DOCX) [file pone.0091116.s005.docx]

### Table S4. Comparison of 1^st^ and 2^nd^ screening of selected 15 samples analyzed by RT-qPCR at CRI (replicated positive samples are shown in bold).

|  |  | **CRI( 1^ST^screening)** | | | | **CRI(verification)** | | | |
| --- | --- | --- | --- | --- | --- | --- | --- | --- | --- |
| **No.** | **Proband** | **Ct [c-ABL]** | **Positivity** | **Ct [p190]** | **Copies** | **Ct [c-ABL]** | **Positivity** | **Ct [p190]** | **Copies** |
| 1. | 44 | 24.79 | 1/3 |  |  | 26.84 | 0/3 |  |  |
| 2. | 166 | 26.68 | 1/3 |  |  |  | 0/3 |  |  |
| 3. | 167 |  | 1/3 | 36.44 | 2 | 26.15 | 0/3 |  |  |
| 4. | 173 | 24.62 | 1/3 | 39.40 | 2 | 26.40 | 0/3 |  |  |
| 5. | **196** |  | **1/3** | 35.69 | 3 | 27.48 | **1/3** | 44.44 | 1 |
| 6. | 200 | 26.68 | 1/3 | 33.59 | 12 | 26.78 | 0/3 |  |  |
| 7. | 250 | 24.38 | 1/3 | 39.49 | 2 | 26.24 | 0/3 |  |  |
| 8. | 251 | 26.19 | 1/3 | 38.08 | 5 | 26.37 | 0/3 |  |  |
| 9 | 257 | 25.35 | 1/3 | 39.17 | 2 | 27.59 | 0/3 |  |  |
| 10. | 259 | 27.3 | 2/3 | 39.19 | 2 | 26.21 | 0/3 |  |  |
|  |  |  |  | 39.85 | 1 |  |  |  |  |
| 11. | 262 | 29.14 | 1/3 | 38.23 | 5 | 26.47 | 0/3 |  |  |
| 12. | 265 | 24.38 | 1/3 | 40.25 | 1 | 26.6 | 0/3 |  |  |
| 13. | **297** |  | **2/3** | 37.06 | 2 | 26.30 | **1/3** | 39.68 | 2 |
|  |  |  |  | 37.37 | 2 |  |  |  |  |
| 14. | **307** |  | **1/3** | 37.90 | 1 | 26.16 | **2/3** | 39.30 | 2 |
|  |  |  |  |  |  |  |  | 40.13 | 1 |
| 15. | **308** | 29.18 | **1/3** | 36.85 | 2 | 23.41 | **2/3** | 42.15 | 1 |
|  |  |  |  |  |  |  |  | 39.78 | 2 |
| Efficiency | | 96% | | 93.0% | | 92.7% | | 96.3% | |
| R2 value | | 0.992 | | 0.993 | | 0.996 | | 0.998 | |
