# Supplementary material for: Incidence of Common Preleukemic Gene Fusions in Umbilical Cord Blood in Slovak Population
Source: PLoS One. 2014 Mar 12;9(3):e91116. doi: 10.1371/journal.pone.0091116 (PMC3951330; doi:10.1371/journal.pone.0091116)
Supplement: Text S1 — Precautionary measures against contamination. (DOCX) [file pone.0091116.s006.docx]

### Text S1. Precautionary measures against contamination.

We have been taking all the necessary measures to avoid contamination during sample handling, processing and analysis. First, MNC from UCB were isolated in
Eurocord-Slovakia, located outside of our Institute. MNC as cell pellets were stored in liquid nitrogen and transported to our N_2_-container before RNA isolation. The total RNA was isolated in a room dedicated only for isolation of RNA from clinical samples and located on 2^nd^ floor, the laboratories dedicated for PCR are located on 3^rd^ floor. PCR mixes were prepared in a separate room (pre-PCR lab) inside of DNA/RNA UV-CLEANER box (Biosan UVT-S-AR) equipped with 2x30W built-in bactericidal lamps and air recirculation.
This UV-box and its equipment (tubes, pipettors, tips, racks, vortex, and microfuge) never came into a contact with template RNA or DNA, or PCR products. The template RNA (for cDNA synthesis) or template cDNA (for multiplex or RT qPCR) was added to PCR mixes in another separate room (PCR lab), again inside of a DNA/RNA UV-CLEANER box (Biosan UVT-B-AR) equipped with 1x25W built-in bactericidal lamp and UV-recirculator. The cDNA was synthesized on PCR cycler, that has been cleaned after each PCR with
DNA–ExitusPlus IF^TM^ (AppliChem), a decontamination solution for the removal of DNA and RNA contaminations, subsequently with 70% ethanol and irradiated with UV-C germicidal lamp (36W) for a minimum of 1 hour (at ~10cm distance). The pipettors containing a filter were wiped with DNA–ExitusPlus IF^TM^ and 70% ethanol and subsequently irradiated on both sides in a crosslinker (Biometra, 254nm, 6x8W tubes) with a dose of 6 Jcm^2^/min. After this procedure, decontaminated pipettors were irradiated inside of PCR box for 30 minutes with their tip ejectors oriented up. Only high-quality filter tips were used exclusively for all pipetting. The loaded tubes or plates for RT qPCR were transferred to a separate rooms (PCR lab 1 and 2), containing RotorGene 2000 and BioRad CFX96, respectively. The PCR products from multiplex PCR were analyzed by electrophoresis in a separate room (post-PCR lab).
